# ETS Transcription Factors in Immune Cells and Immune-Related Diseases

**DOI:** 10.3390/ijms251810004

**Published:** 2024-09-17

**Authors:** Yaxu Yang, Xue Han, Lijun Sun, Fangyu Shao, Yue Yin, Weizhen Zhang

**Affiliations:** 1 Department of Physiology and Pathophysiology, State Key Laboratory of Vascular Homeostasis and Remodeling, School of Basic Medical Sciences, Peking University, Beijing 100191, China; 1810305332@pku.edu.cn (Y.Y.); sunlj9002@163.com (L.S.); 2 Department of Pharmacology, State Key Laboratory of Vascular Homeostasis and Remodeling, School of Basic Medical Sciences, Peking University, Beijing 100191, China; hanxuehx313@163.com (X.H.); shaofangyu@bjmu.edu.cn (F.S.)

**Keywords:** ETS family, transcription factor, immune cell, immune-related diseases, PU.1, ETV5

## Abstract

The development, differentiation, and function of immune cells are precisely regulated by transcription factors. The E26 transformation-specific (ETS) transcription factor family is involved in various physiological and pathological processes by regulating cell proliferation, differentiation, and apoptosis. Emerging evidence has suggested that ETS family proteins are intimately involved in the development and function of immune cells. This review summarizes the role of the ETS family in immune cells and immune-related disorders. Seven transcription factors within the ETS family, including PU.1, ETV5, ETV6, ETS1/2, ELK3, and ELF1, play essential roles in the development and function of T cells, B cells, macrophages, neutrophils, and dendritic cells. Furthermore, they are involved in the occurrence and development of immune-related diseases, including tumors, allergies, autoimmune diseases, and arteriosclerosis. This review is conducive to a comprehensive overview of the role of the ETS family in immune cells, and thus is informative for the development of novel therapeutic strategies targeting the ETS family for immune-related diseases.

## 1. Introduction

Immune cells are those involved in the immune system, responsible for recognizing and destroying pathogens, removing waste, initiating and regulating immune responses, and maintaining overall health. According to surface markers and functional characteristics, immune cells are divided into T lymphocytes, B lymphocytes, macrophages, neutrophils, dendritic cells, and others. The expression of specific genes within cells, the local immune microenvironment, and the interactions among immune cells collectively modulate the development and function of immune cells and contribute to the onset and progression of diseases [1,2]. Among them, transcription factors exert crucial roles in the development and function of immune cells by regulating key genes. 

The E26 transformation-specific (ETS) family is highly evolutionarily conserved [3]. The ETS family consists of 28 genes in humans, while it consists of 27 genes in mice [4]. Based on the differences in sequences and sequence preferences, the ETS family is divided into four classes and 12 subfamilies [5,6,7,8]. The ETS family is involved in a variety of physiological processes, including embryonic development, cell cycle, differentiation, proliferation, apoptosis, migration, and secretion [9,10,11,12,13,14]. In recent years, much attention has been focused on the role of the ETS family in the development and differentiation of immune cells.

In this review, we summarize the role of the ETS family in various immune cells and immune-related diseases. This knowledge contributes to the systematic understanding of the pathogenesis and physiology of ETS factors in immune cells and provides novel strategies for intervention in immune cell-mediated diseases.

### 1.1. ETS Family Profile

**Figure 1:**
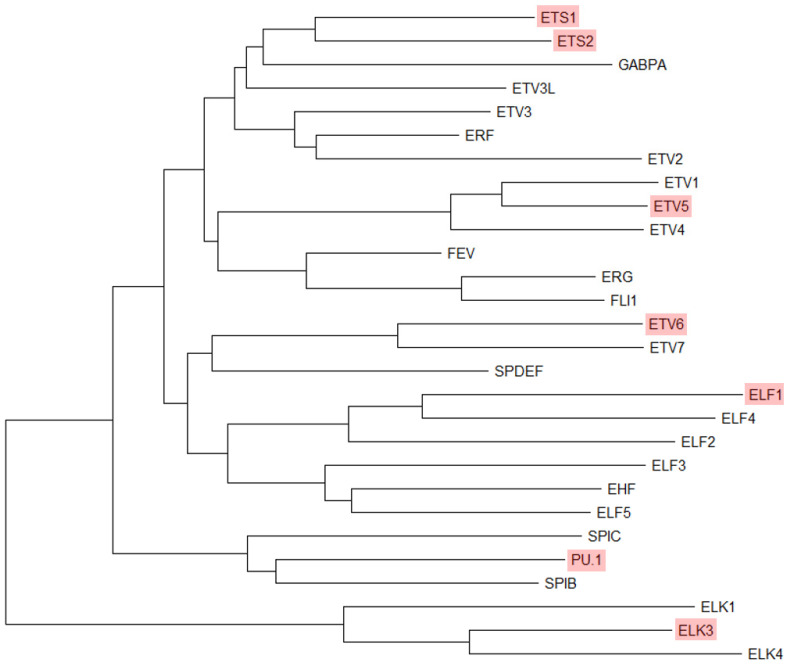
Homology tree of human ETS transcription factors. ETS factors mentioned in this review are shown in red. Neighbor-Joining test and MEGA11 software were used for the protein sequence alignment.

The ETS family is evolutionarily highly conserved and contains multiple transcription factors [3]. ETS family proteins all contain a conserved sequence region known as the ETS domain. This is a winged helix-turn-helix (wHTH) DNA-binding domain consisting of 85 amino acids that form three α-helices and a four-stranded β-sheet, which recognizes the core GGAA/T sequence (ETS binding site) [15]. The third α helix establishes the main groove contact with DNA (GGAA/T sequence), and the different affinities of the sequences flanking the core motif determine the DNA recognition sequence preferences of several family members [3]. In addition, approximately half of the human ETS family members also contain the N-terminal pointed domain, which consists of 65 to 85 amino acids [16]. Based on the differences in sequence, the ETS family is divided into 12 subfamilies: ETS, ERG, PEA3, ETV2, TCF, ERF, PDEF, ELF, ESE, TEL, SPI, and ELG [8]. Based on sequence preference, the ETS family is also divided into four categories (Figure 1). Class I contains over half of the ETS family (the ETS, ERG, PEA3, ETV2, TCF, ERF, and ELG subfamilies), showing the same consensus sequence (ACCGGAAGT) [17]. Class II contains the TEL, ESE, and ELF subfamilies, which differ on the first nucleotide (CCCGGAAGT) [17]. Class III is composed of the SPI subfamily and has a preference for adenine-rich sequence binding sites [5]. Class IV only includes PDEF, which prefers the GGAT sequence rather than GGAA [6]. 

**Figure 2:**
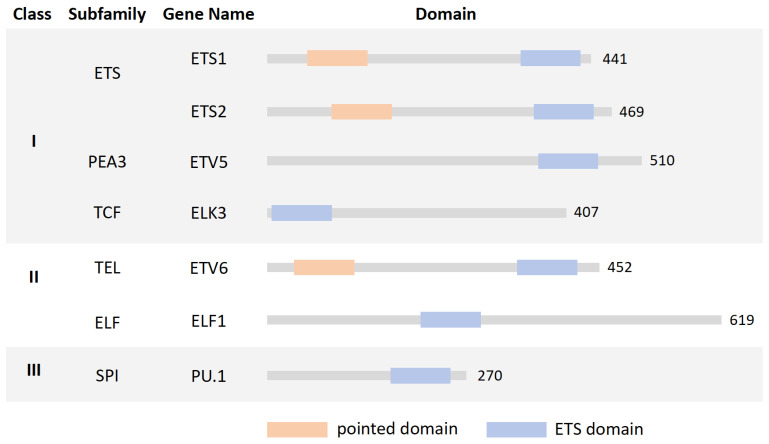
ETS family’s involvement in immune cells. Figure 2 was drawn by Figdraw.

**Figure 3:**
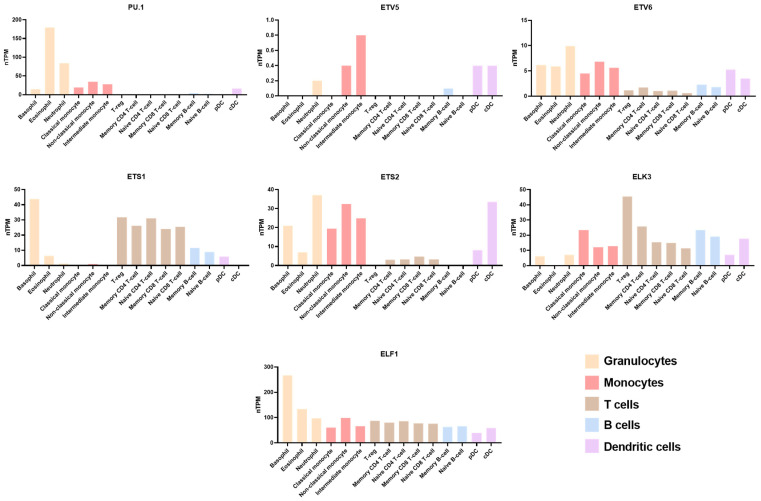
The expression of ETS transcription factors in immune cells. PU.1 available from https://www.proteinatlas.org/ENSG00000066336-SPI1/immune+cell. ETV5 available from https://www.proteinatlas.org/ENSG00000244405-ETV5/immune+cell. ETV6 available from https://www.proteinatlas.org/ENSG00000139083-ETV6/immune+cell. ETS1 available from https://www.proteinatlas.org/ENSG00000134954-ETS1/immune+cell. ETS2 available from https://www.proteinatlas.org/ENSG00000157557-ETS2/immune+cell. ELK3 available from https://www.proteinatlas.org/ENSG00000111145-ELK3/immune+cell. ELF1 available from https://www.proteinatlas.org/ENSG00000120690-ELF1/immune+cell (all accessed on 14 September 2024).

Through literature searches, we found that seven ETS family members, including PU.1, ETS variant transcription factor (ETV) 5 and 6, ETS Proto-Oncogene 1 and 2 (ETS1 and 2), ELK3, and E74 Like ETS transcription factor 1 (ELF1), play roles in the development and differentiation of immune cells. The subfamily and their pointed domain and ETS domain are shown in Figure 2. PU.1 pertains to the class III SPI subfamily and is encoded by the Spi1 gene. PU.1 is specifically expressed in hematopoietic lineage cells such as macrophages, granulocytes, and B lymphocytes [18]. ETV5 belonging to the Class I PEA3 subfamily, and also named ETS-related molecule (ERM), is widely expressed in multiple organs and shows high expression levels in immune cells [19]. ETV6 is one of the class II TEL subfamilies and encodes a transcriptional repressor, exerting a pivotal role in hematopoiesis and embryonic development [20]. The other four transcription factors are class I or II: ETS1/2, ELK3, and ELF1, which pertain to the ETS subfamily, the TCF subfamily, and the ELF subfamily, respectively. The expression of ETS family factors varies greatly among different immune cells, which may suggest that they have different roles in different immune cells (Figure 3).

## 2. ETS Family in Immune Cells

### 2.1. T Cell

T lymphocytes differentiate and mature in the thymus, then circulate through the bloodstream to peripheral lymphoid organs where they settle and proliferate. Upon activation by antigens, T cells differentiate and proliferate into functionally heterogeneous effector cells. Among these, helper T (Th) lymphocytes, including Th1, Th2, Th9, and Th17, regulate other lymphocytes in performing their functions. Signal transducer and activator of transcription (STAT) signaling pathways respond to different cytokines to induce different subtypes of Th cells. Transcription factors STAT4 and STAT6 mediate the induction of Th1 and Th2 phenotypes by IL-12 and IL-4. IL-6, IL-21, and IL-23 can activate STAT3, inducing the differentiation and maintenance of Th17 cells. Th9 cells, which secrete IL-9 and IL-21, are differentiated depending on IL-4 and TGF-β [21]. 

ETV5 is expressed at its highest level in γδ thymocytes expressing the Vγ2 T cell receptor (TCR) chain. T cell-specific deletion of ETV5 does not affect the total number of γδT cells, but results in fewer mature Vγ2 thymocytes and more naive cells [19]. In IL-6- and IL-23-stimulated Th17 cells, STAT3 directly binds to the ETV5 promoter to activate the expression of ETV5. ETV5 can recruit histone acetyltransferase p300 at the IL17a-IL17f sites, resulting in a reduction in repressive histone marks (H3K4 methylation and H3K27 acetylation) and an increase in active histone marks (H3K27 methylation). This alteration directly facilitates the expression of IL-17a and IL-17f [22]. ETV5 also directly binds to conserved non-coding sequences of the IL-10 sites to promote the production of IL-10 by Th2 cells [23]. Furthermore, overexpression of ETV5 enhances the level of IFN-γ in Th1 cells. Nevertheless, the expression of ETV5 in STAT4-deficient T cells is insufficient to restore IFN-γ to a normal Th1 level, indicating that ETV5 may not be the only transcription factor induced by IL-12 [24].

PU.1 promotes or inhibits gene expression in early T cells through two independent mechanisms. It promotes gene expression by opening chromatin and cooperating with transcriptional cofactor nucleation complexes, such as SATB1 and RUNX1. Conversely, it can also indirectly inhibit T-cell gene expression by competitively redistributing its cofactors [25]. PU.1 can rapidly induce transposase accessibility and recruit histone acetyltransferases [26]. PU.1 regulates Th9 cell differentiation by controlling unique and dynamic epigenetic modifications in the promoter, such as inhibiting histone methylation to induce Th9-specific PU.1 expression [27]. PU.1 increases histone acetylation at the IL-9 sites through direct interaction with histone acetyltransferases, thereby promoting IL-9 expression [28]. However, studies have also found that PU.1 inhibits Th9 cell differentiation and reduces IL-9 production by Th9 cells through selective autophagy [29]. In addition, ETV5 and PU.1 can also act synergistically. ETV5 promotes the production of IL-9 in Th9 cells by recruiting histone acetyltransferases to different IL-9 binding sites [30].

ELF1 is highly expressed in T cells and has been reported to regulate the expression of multiple T cell-related genes, including granulocyte-macrophage colony-stimulating factor (GM-CSF), interleukin-2 receptor α subunit, and CD4 [31]. The Ets1 binding site of the GM-CSF promoter is crucial for the synergy between acute myeloid leukemia 1 (AML1) and calmodulin-dependent protein phosphatase calcineurin in Jurkat T cells. Under the stimulation of TCR, both subunits of activated calcineurin interact directly with the N-terminal homologous domain portion of AML1, which is an essential transcription factor for hematopoiesis [32]. AML1 recruits calcineurin phosphatase at the GM-CSF promoter, targeting glycogen synthase kinase-3β-phosphorylated ETS1 [33].

### 2.2. B Cell

B lymphocytes play a critical role in adaptive immunity by producing high-affinity antibodies (immunoglobulins), generating immune memory, acting as antigen-presenting cells, and secreting cytokines. The development of B lymphocytes is a multi-step process involving the ordered expression of intracellular and surface-localized markers and the rearrangement of immunoglobulin gene segments. The process involves a range of transcription factors (such as PU.1) and cell surface molecules (such as CD20) [34].

B lymphocytes were deficient in PU.1 deficient mice, suggesting that PU.1 is an indispensable regulator of B-cell development [35]. PU.1 can bind to interferon regulatory factor (IRF) 4 and IRF8 to regulate the class switching and recombination of immunoglobulins [36]. PU.1 regulates early B-cell development by regulating the cell receptor signaling pathway of B cells, including the CD40L receptor, Toll-like receptor ligands [37], and IL-7Ra [38]. The histone acetyltransferase CREB-binding protein and EP300 can enhance the binding ability of PU.1 to the CD20 promoter by mediating the acetylation of PU.1, thus promoting CD20 expression and B-cell development [39]. On the contrary, the binding of the Spi-C transcription factor to PU.1 induces the DNA binding of PU.1, which reduces the DNA binding ability of PU.1, thus inhibiting the expression of genes related to B-cell development [40].

### 2.3. Macrophage

Macrophages are involved in the recognition, phagocytosis, and degradation of cell debris and pathogens. These cells exert pro-inflammatory or anti-inflammatory effects through the release of distinct cytokines and chemokines.

The expression of monocyte/macrophage-specific markers CD 11b and F4/80 in fetal liver cells and bone marrow-derived macrophages is dependent on the presence of functional PU.1 [41]. The PEST (a region rich in proline, glutamate, serine, and threonine) sequence of the PU.1 protein is an important domain for protein–protein interaction in B cells and macrophages [42]. Expression of the PEST-binding domain of PU.1 in macrophages results in reduced proliferation [43]. During macrophage differentiation, PU.1 keeps macrophage-specific genes accessible by preventing the polycomb repressive complex 2 from binding to transcriptional regulatory elements [44]. Lipopolysaccharides (LPS) treatment induces the formation of a complex between PU.1 and c-Jun, while the elimination of the complex inhibits the transcriptional activity of PU.1 on gene expression [45]. NF-κB-induced kinases regulate the expression of the COX-2 gene by inducing the phosphorylation of PU.1, thereby participating in the inflammatory response and polarization of macrophages [46,47,48]. 

ETS1 inhibits the expression of the IL-1β-induced MUC5AC gene by disrupting the interaction of NF-κB and CREB on the MUC5AC promoter, thus alleviating lung infection in mice [49]. By inhibiting MAPK/NF-κB signaling, ETS2 directly binds to promoters, inhibits transcription, reduces the production of IL-6, TNF-α, and IFN-β in macrophages, and alleviates LPS-induced inflammation [50]. Endogenous expression of ELK-3 is inversely proportional to NOS2 and significantly inhibits NOS2 promoters in a dose-dependent manner, attenuating endogenous NOS2 in induced inflammatory responses [51]. ELK-3 is an important inhibitory factor for the transcription of the HO-1 gene, contributing to the strict control of the regulation of the HO-1 gene under inflammatory stimulation [52].

During bacterial infection, such as *M.avium*, activation of the RNA-sensing pathway in macrophages inhibits the E3 ubiquitin ligase CRL4COP1/DET1 and stimulates the expression of intercellular cell adhesion molecule-1 (ICAM-1) [53]. ICAM-1 is required for the formation of immune synapses between infected macrophages and antigen-specific CD4+ T cells [54]. CRL4 targets the transcription factor ETV5, which is degraded by the ubiquitin–proteasome system. In the absence of ETV5, ICAM-1 expression is significantly reduced [54]. Moreover, PU.1 and ETS2 significantly enhance the activation of BCL-x, which is essential for the cytotoxic function and survival of macrophages [55]. During E.coli infection, LPS decreases the promoter activity of EIK-3, thereby facilitating the phagocytic capacity of macrophages [56].

In addition to initiating immune and inflammatory responses, the ETS family is also involved in the metabolic regulation of macrophages. The expression of ETV5 is significantly reduced in adipose tissue macrophages of obese mice, which leads to macrophage activation and inhibition of endoplasmic reticulum stress, and IL6 expression in macrophages [57]. In addition, when expressed at macrophage levels in mature adipocytes, PU.1 represses genes with nearby adipocyte-specific PPARγ binding sites, while a common macrophage-adipocyte gene expression program is retained, suggesting a cell-remodeling function of PU.1 in terminally differentiated adipocytes [58].

### 2.4. Neutrophil

As the principal immune cells in peripheral blood, neutrophils constitute the first line of defense against fungal and bacterial infections and serve as a significant regulator of the adaptive immune system [59]. PU.1 can be involved in neutrophil differentiation by activating its transcription by binding to promoters of microtubule-associated protein 1S and death-associated protein kinase 2 [60], and by directly activating the transcription of hexokinase 3 [61]. PU.1 also promotes neutrophil nuclear division by regulating the transcription of laminin B receptor, an intracapsular protein that is necessary for neutrophil nuclear segmentation [62]. PU.1 can also suppress neutrophil activation by regulating the inflammatory epigenome of neutrophils. PU.1 inhibits enhancer accessibility through the recruitment of histone deacetylases, thereby preventing JUNB, a key mediator of inflammatory activation, from accessing chromatin [63]. Additionally, through forward genetic screening of zebrafish, it has been identified that the conserved PU.1-ZBTB11-TP53 pathway between fish and mammals is a regulator of neutrophil development [64]. 

### 2.5. Dendritic Cells

Dendritic cells (DCs), the most antigen-presenting cells, can recognize and respond to pathogen-associated and risk-related signals to stimulate T cells to initiate adaptive immunity [65]. DC subsets are divided into two major lineages: plasmacytoid dendritic cells (pDCs), derived from lymphoid stem cells; and conventional DCs (cDCs), differentiated from myeloid stem cells under the stimulation of GM-CSF, known as myeloid DCs [66]. Major histocompatibility complex class II molecules (MHCII) and integrin CD11c (encoded by ITGAX) are highly expressed in cDCs [67,68]. MHCII expression is regulated by Class II trans-activator (CIITA), a cofactor with three distinct promoters, pI, pIII, and pIV [69]. pI and pIII play major roles in cDCs and pDCs, respectively [70].

PU.1 is differentially expressed in DC, with high expression in cDCs and low expression in pDCs [71]. Moreover, PU.1 can induce precursor T cells to become cDCs [72], whereas hematopoietic progenitors of PU.1-deficient mice cannot produce cDCs in vitro [73]. PU.1 activates the ITGAX promoter by directly binding to the cis-acting element, participating in the transcription of CD11c and subsequent protein expression [74]. PU.1 independently binds to one side of the CIITA pI promoter and concurrently forms a heterodimer with IRF8 to bind to another site [70]. PU.1 also promotes CIITA pI expression by regulating histone acetylation in the CIITA pI promoter region [75]. Although the expression level of PU.1 in pDCs is low, it can still activate pIII by directly binding to the CIITA pIII promoter [76].

PU.1 is also involved in the regulation of other key genes governing DC functions, such as CD80, CD86 [77], OX40L [78], CCL22 [79], CCR7 [80], and DC-SIGN [81,82]. CD80, CD86, and OX40L contribute to antigen presentation and thus are crucial for activation of T cells [77,78]. Chemokine CCL22 acts on CCR4-expressing cells, including Th2 and Treg [83]. CCR7 mediates the entry of dendritic cells into the T-cell zones in lymph nodes, initiating antigen presentation and T-cell responses [84]. Dendritic cell-specific intercellular adhesion molecule-3-grabbing nonintegrin (DC-SIGN) mediates antigen capture, processing, and presentation [85]. PU.1 initiates transcription by binding them to their promoters, which in turn perform their respective functions.

### 2.6. Other Immune Cells

A few studies have reported the function of the ETS family in other immune cells (mast cells, eosinophils, and basophils). Interestingly, PU.1 and other transcription factors are highly expressed in eosinophils, but there are few studies on them.

Mast cells (MCs) exert a crucial role in IgE-mediated allergic reactions. When PU.1 is downregulated in bone marrow-derived MCs (BMMCs), the IgE-mediated activation is attenuated. In BMMCs, knocking down PU.1 reduces IgE-mediated activation [86]. Overexpression of PU.1 induces the expression of several monocyte-specific genes (MHC II class, CD11b, CD11c, and F4/80) on BMMCs cultured in vitro [87]. PU.1 directly binds to the CIITA pIV promoter to activate pIV in mast cells [87]. Furthermore, the human IL1RL1/ST2 gene encodes the IL-33 receptor, with mast cells and basophils being the main targets of IL-33 [88]. In human mast cell line LAD2 and basophilic cell line KU812, PU.1 directly activates the ST2 promoter through ETS-family-related cis-elements [89]. In addition, PU.1 and ETS-1 play an important role in the specific gene expression of mast cells or eosinophils in conjunction with GATA-1 or GATA-2. In monocytes and red blood cells, PU.1 is antagonistic to GATA-1/2 to regulate cell-type-specific gene expression [90]. However, in mast cells and eosinophils, PU.1 positively regulates GATA-1 or GATA-2, and both of them are essential during development [91,92].

## 3. ETS in Immune-Related Diseases

### 3.1. Cancer

Tumor-associated macrophages are important immune cells that constitute the tumor microenvironment and participate in regulating various complex immune responses of tumors [93]. The activation of the BTK signaling pathway induced by PU.1 leads to the activation of the AKT/ mTOR pathway in macrophages, which promotes tumor migration, invasion, and proliferation [94]. Tumor-infiltrating lymphocytes are objective predictors of the status of cancer sentinel lymph nodes and survival rates. In colon cancer, ETV1 and ETV5 expression levels are positively correlated with infiltration of CD8+ T cells, CD4+ T cells, macrophages, DCs, and cancer-associated fibroblasts [95]. Also, ETV6 allelic mutations are associated with lymphoma [20]. In acute lymphoblastic leukemia (ALL), ETV6-RUNX1 is the most common translocation [96,97]. Insulin-like growth factor 2 binding protein 1 is overexpressed in ETV6-RUNX1 positive B-cell -ALL and drives carcinogenic signaling [98].

The abnormal expression and chromosomal translocations of FLI1, ERG, ETV6, and PU.1 are often associated with leukemia [99]. In addition, the research of the ETS family in cancer is mostly focused on the proliferation, apoptosis, migration, and angiogenesis of cancer cells [8]. Other parts related to immune cells lack relevant research.

### 3.2. Allergies and Autoimmune Diseases

PU.1 promotes the polarization of macrophages and induces the expression of the allergenic factors YM-1 and FIZZ-1, which are involved in the development of allergic inflammation [100]. The expression of ETV5 in intestinal mucosa and peripheral blood CD4 T cells of inflammatory bowel disease (IBD) patients is significantly increased. Overexpression of ETV5 enhances the Th1/Th17 immune response and induces intestinal mucosal inflammation by up-regulating the phosphorylation of STAT3 and STAT4 [101]. In the 2,4,6-trinitrobenzene sulfonic acid solution-induced IBD mice, the specific deletion of ETV5 in CD4+ T cells improves intestinal inflammation and fibrosis [102]. Follicular helper T (Tfh) cells can produce high-affinity antibodies against specific pathogens, but their overproduction is associated with systemic autoimmune diseases such as systemic lupus erythematosus (SLE) [103]. The transcriptional suppressor capicua maintains immune tolerance by inhibiting abnormal activation of adaptive immunity [104]. ETV5 inhibits excessive enhancement of Tfh cell differentiation in capicua-deficient CD4+ T cells [105]. T cell-specific deletion of ETV5 improves cell differentiation and autoimmune phenotypes in the SLE mice. SPP1 is an ETV5 target that promotes the differentiation of Tfh cells. The ETV5 and SPP1 levels of CD4+ T cells are increased in SLE patients and positively correlated with disease activity [106]. 

### 3.3. Arteriosclerosis 

Macrophages play a crucial role in plaque development, local inflammation, and thrombosis. In a mouse model of arteriosclerosis, ETV6 expression is elevated in aortic macrophages. By inhibiting the phosphorylation of IKKβ and NF-κB p65, ETV6 knockout inhibits the expression of inflammatory factors such as IL-1β, IL-6, and TNF-α, thereby reducing macrophage-mediated inflammation [107].

## 4. Conclusions

**Figure 4:**
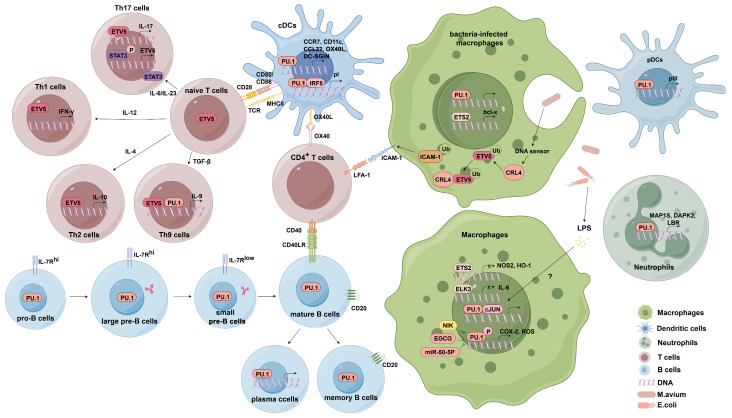
ETS family in immune cells. In Th17 cells, STAT3 directly binds to the ETV5 promoter to activate the expression of ETV5. ETV5 directly facilitates the expression of IL-17a and IL-17f. In Th2 cells, EVT5 promotes the production of IL-10. In Th1 cells, ETV5 enhances the level of IFN-γ. In Th9 cells, ETV5 and PU.1 promote the production of IL-9. PU.1 regulates early B-cell development and differentiation. In macrophages, ELK3 inhibits the expression of NOS2 and HO-1. ETS2 inhibits the expression of IL-6. Phosphorylated PU.1 initiates transcription of COX2 and ROS. LPS induces the formation of a complex between PU.1 and c-Jun promoting transcription. In bacteria-infected macrophages, PU.1 and ETS2 significantly enhance the activation of BCL-x. CRL4 targets the ETV5, which is degraded by the ubiquitin–proteasome system. In neutrophils, PU.1 activates the transcription of MRP1S and DAPK2. PU.1 also promotes neutrophil nuclear division by regulating the transcription of LBR. In cDCs, PU.1 activates the transcription of CCR7, CD11c, OX40L, DC-SIGN, and CIITA pI and forms a heterodimer with IRF8 promoting CIITA pI expression. In pDCs, PU.1 activates pIII by directly binding to the CIITA pIII promoter. Scheme 1. HO-1; Cyclooxygenase 2, COX2; Reactive oxygen species, ROS; Lipopolysaccharides, LPS; B-cell lymphoma, BCL; Cullin-RING E3 ubiquitin ligases, CRL4; Microtubule-associated protein 1S, MRP1S; Death-associated protein kinase 2, DAPK2; Laminin B receptor, LBR; Conventional DC, cDC; Plasmacytoid dendritic, pDC; C-C Motif Chemokine Receptor 7, CCR7; OX40 ligand, OX40L; Dendritic cell-specific ICAM-grabbing non-integrin, DC-SIGN; Interferon regulatory factor 8, IRF8; Class II trans-activator, CIITA.

This review summarizes our current understanding of ETS family transcription factors on immune cells and immune-related diseases (Figure 4). To date, seven transcription factors in the ETS family have been identified to be involved in the regulation of immune cells. Among these, PU.1 and ETV5 are the most studied. PU.1 and ETV5 not only participate in the development and differentiation of a variety of immune cells such as T cells, B cells, macrophages, neutrophils, and dendritic cells, but also affect key genes that control infection and inflammation. Deficiency of PU.1 and ETV5 can lead to developmental defects and functional abnormalities of immune cells. Immune-related diseases, including cancer, allergies, autoimmune diseases, and atherosclerosis, are often associated with abnormal expression of PU.1 and ETV5.

Despite the fact that the role of the ETS family in immune cells has been partially elucidated in recent years, their complex role remains to be revealed. First, the ETS family has a large number of members, and the effects of the members, other than the seven members that have been studied so far, on immunity are unknown. Second, the ETS family members may exhibit opposite effects on different immune cells. There may be synergistic or antagonistic regulatory effects within the ETS family or with other transcription factors. Third, the expression levels of different ETS members vary over time and space during immune cell development and differentiation.

## 5. Future Directions

Although these complex roles may be beneficial in balancing the intensity of the immune response, they greatly increase the difficulty of research. In forthcoming research, on the one hand, it is essential to thoroughly analyze the correlation between the ETS family and various diseases and disease processes based on clinical samples. On the other hand, before studying the immune microenvironment, it is necessary to clarify the roles and mechanisms of the ETS family in individual immune cells using immune cell-specific transgenic animals.
